# Delayed presentation of familial intestinal malrotation with volvulus in two adult siblings

**DOI:** 10.1308/003588412X13373405384819

**Published:** 2012-09

**Authors:** J Nath, AP Corder

**Affiliations:** Wye Valley NHS Trust,UK

**Keywords:** Intestinal malrotation, Genetics, Midgut volvulus

## Abstract

Intestinal malrotation is an uncommon cause of abdominal pain and normally presents during infancy. Familial cases of malrotation are extremely rare in the absence of other congenital malformations. We present the case of a 22-year-old woman with undiagnosed chronic abdominal pain and her previously well 16-year-old brother who presented within 18 months of each other with acute midgut volvulus secondary to intestinal malrotation. Clinicians should be aware of this rare but serious cause of abdominal pain.

Intestinal malrotation is a well recognised cause of abdominal pain in children with a described incidence of 1 in 500 live births.^1,2^ Malrotation occurs due to failure of the normal embryological rotation of the developing gastrointestinal tract around the superior mesenteric artery and can vary in severity. The surgical management of malrotation is well described and includes Ladd’s procedure or a more anatomical repositioning of the abdominal viscera.^3^

The majority of patients with malrotation present in the neonatal period with bilious vomiting and a diagnosis can be confirmed with barium studies.^4,5^ Symptoms associated with later presentation are less specific and include chronic pain and diarrhoea. Such non-specific symptoms can cause significant difficulty in diagnosis and the true incidence of intestinal malrotation in adults is not known^6^ with many probably being labelled as having ‘functional bowel disorder’. Midgut volvulus can occur as a complication of malrotation and can in turn cause obstruction and compromise bowel vascularity. Such diagnoses are often only made at laparotomy.

Cases of familial intestinal malrotation are rare and normally occur in conjunction with other genetic abnormalities including gut atresias, biliary, cardiac and pancreatic malformations.^5,7^ However, isolated malrotation has been described in several families with an autosomal dominant inheritance pattern although no single genetic abnormality has been identified.^8,9^

## Case history

A 22-year-old woman presented to our department with severe abdominal pain shortly after being discharged from another hospital. She had a long history of abdominal pain and had seen multiple paediatricians and gastroenterologists. Over the following few days her clinical condition deteriorated and she developed peritoneal signs and tachycardia. Urgent computed tomography (CT) was performed, which demonstrated the presence of midgut volvulus with an occluded superior mesenteric artery. The patient proceeded to emergency laparotomy. This revealed intestinal malrotation and a midgut volvulus. Unfortunately, there was a large amount of necrotic small bowel with a combined total of 15cm of viable proximal jejunum and terminal ileum.

Following a subtotal small bowel resection and mucous fistula formation, a second look laparotomy was performed the following day and gut integrity was restored. Recovery was complicated by the development of several intra-abdominal collections treated with radiological drainage and antibiotics. The patient is well and gaining weight 18 months after discharge although she remains on long-term cyclical parenteral feeding.

**Figure 1:**
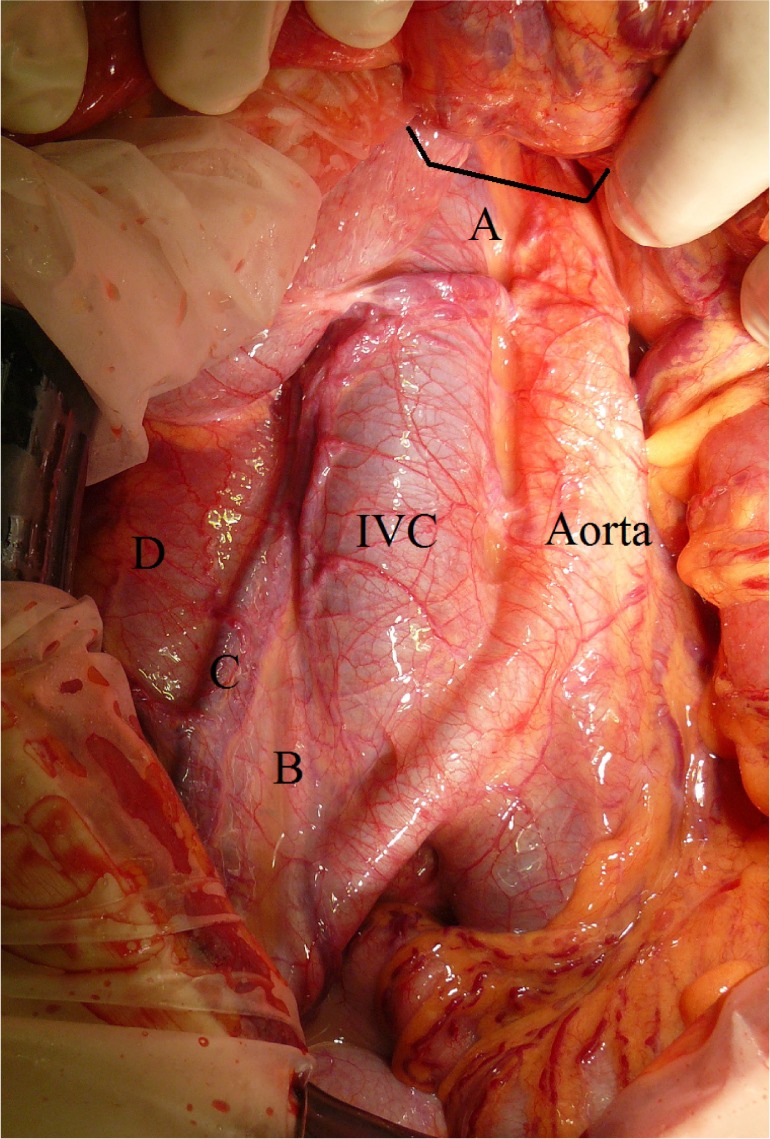
Intra-operative photograph: A = root of small bowel mesentery; B = right ureter; C = gonadal vessels; D =psoas muscle; IVC = inferior vena cava

Fifteen months later, this patient’s sixteen-year-old brother presented with a short history of sudden onset central abdominal pain. He had no prodromal symptoms. Although he displayed no evidence of peritonism and his pain had settled somewhat overnight, CT was performed and confirmed volvulus. At laparotomy he was found to have intestinal malrotation with midgut volvulus. Although the bowel was cyanosed, this promptly improved following untwisting of the volvulus and no bowel resection was necessary. As with his sister, the caecum was in the left upper quadrant, the duodenum was intraperitoneal and there was only a very narrow attachment of small bowel mesentery to the posterior abdominal wall around which the volvulus had occurred (Fig 1).

The Ladd’s bands were divided, the bowel was untwisted and replaced in the anatomical position with anchoring of the small bowel mesentery to the posterior abdominal wall. This patient made a steady post-operative recovery, which was complicated by a pneumonia. He remains well.

## Discussion

To our knowledge this is the first report of familial malrotation presenting with acute volvulus in adulthood in siblings with no other syndromic abnormalities. Studies have identified the transcription factor FOXF1 as playing a key role in the formation of the dorsal mesentery^7^ and both of these patients had a narrow attachment of mesentery to the posterior abdominal wall, around which the volvulus had occurred.

The diagnosis of intestinal malrotation can be difficult in young adults as symptoms are often vague and remitting. The lady in this report was seen on an outpatient basis by both paediatricians and gastroenterologists multiple times as a child and young adult and had been labelled as having functional bowel disorder. She had at least three admissions to hospital with acute self-limiting abdominal pain and ultrasonography had confirmed the presence of a small gallbladder polyp, further confusing the diagnostic picture. She had also undergone a barium swallow as a neonate following episodes of vomiting, which was reported as normal. All of these factors, coupled with a reluctance to expose young adults to the radiation associated with imaging such as CT, led to the delay in treatment to the point where extensive bowel necrosis had occurred.

Fortunately, when her brother presented with similar symptoms, CT was performed early even though his symptoms had seemingly improved. This undoubtedly led to a quicker diagnosis in his case, which obviated the need for intestinal resection.

## Conclusions

Clearly, CT of all patients with self-remitting abdominal pain would be both unworkable and hazardous. Acute midgut volvulus in patients with malrotation can cause severe abdominal pain and, where spontaneous untwisting occurs, symptoms may be self-limiting. As ever, one should be cautious in accepting a diagnosis of functional bowel disorder in patients with known chronic symptoms that present acutely. When symptoms do not settle in such patients, an alternative explanation must be sought. Familial malrotation is rare in patients in the absence of other syndromic abnormalities and we would not recommend routine screening for family members of those affected. However, a diagnosis of malrotation with volvulus should be considered if a patient with a family history of midgut malrotation presents with abdominal pain.
